# Wounds of the Eye and Their Treatment*Read before the Medical Association of Georgia, Savannah, Ga., April 17, 1895.

**Published:** 1895-06

**Authors:** T. E. Mitchell

**Affiliations:** Columbus, Ga.


### Southern Medical Record.

A MONTHLY JOURNAL OF MEDICINE AND SURGERY.

VOL. XXV. ATLANTA, GA. JUNE, 1895. No. 6.

### Original Articles.

### WOUNDS OF THE EYE AND THEIR TREATMENT.*

BY T. E. MITCHELL, M. D., COLUMBUS, GA.

GENTLEMEN—After accepting the invitation of the programme committee to read a paper at this meeting of the association, I naturally began to cast about for a subject, and, in doing so, no one presented itself to my mind that would likely be of more interest to the general practitioner than Wounds of the Eye and Their Treatment. The subject is one of large proportions, and one, too, upon which much might be written were it handled exhaustively.

I shall treat the topic, however, in a somewhat superficial manner, presenting it in as practical a way as possible consistent with the amount of time that I propose to consume.

I shall make no attempt at originality of thought or go minutely into the technique of diagnosis or treatment and shall presume that you are familiar with the anatomy and physiology of the eye and its appendages, the lids.

Little more than reference will be made to sympathetic inflammation, that much-to-be-dreaded complication or sequela of traumatic iridocyclitis.

A wound, as we shall consider it, consists of a solution of continuity of the eye or its appendages, produced either directly

*Read before the Medical Association of Georgia, Savannah, Ga., April 17, 1895.

or indirectly by sudden mechanical force or by the destructive properties of some chemical agent as acids or alkalies, generally the latter in the form of lime, either slaked or unslaked.

For convenience of description, we may divide wounds of the eye, as in other parts of the body, into incised, punctured, lacerated and contused wounds. Another classification would be into aseptic and septic wounds. So likewise we may have a simple, compound, or complicated wound. Simple when the agent producing the wound and any other foreign substance carried by it into the rent is removed, and complicated when any foreign body, whether the missile inflicting the wound or material carried by it, remains in the injured member. A compound wound, we may say, is one in which two or more of the integral parts of the eye sustain a simultaneous injury. Thus we may have a punctured wound of the cornea with an escape of aqueous and a lacerated and prolapsed condition of the iris. Indeed, compound wounds are more frequently met with than any other variety. Our prognosis will depend largely upon the seat of injury, and, upon its being simple or complicated. A simple wound, or a complicated one that is readily converted into a simple one may often offer a favorable prognosis though there be considerable laceration of tissue, provided the injury be not in the ciliary region. That part of the eyeball comprising a zone extending from the sclero-corneal junction in front, posteriorly six millimeters, and known as the ciliary region, has been very properly called by Nettleship the zone of danger, owing to the fact that wounds in this region are most apt to set up an intractable inflammation resulting in panophthalmitis with total destruction of the eye, and, by sympathetic involvement, its fellow of the opposite side as well. This is due to the anatomical fact that in this zone of danger is situated the ciliary body, composed almost entirely of blood-vessels, and the ciliary nerves as well as the peripheral attachment of the iris.

Those fundamental principles laid down for treatment of wounds in other parts of the body hold good here: (1) arrest hemorrhage, this will rarely be found troublesome; (2) mitigate any existing shock; (3) cleanse the wound of every vestige of foreign matter; (4) render the wound absolutely aseptic by thorough irrigation with an antiseptic solution

(corrosive sublimate 1 to 2,000—5,000); (5) coaptation of the severed parts, by sutures if necessary; (6) antiseptic dressing and (7) position and rest, which latter we accomplish with atropia or scopolamine, two to five grains to water one ounce, and a light pressure bandage to control the movements of the lids.

We shall now speak of wounds of individual parts of the eye. Contused wounds of the eye, of which an ordinary black eye is a familiar type, are caused by blunt instruments; as for instance, the closed fist of a combatant, and consist of extravasated blood (ecchymosis) from subcutaneous hæmorrhage and an infiltration of serum (œdema). If seen early, apply cold for several hours to lessen the effusion, after this, warm applications, evaporating lotions such as lead water and laudanum, ammonium chloride, five grains to alcohol one ounce, are indicated. Absorption of the effused material will usually take place in from seven to fourteen days, leaving the eye physically and functionally unimpaired.

Incised wounds of the lids require careful coaptation of the divided tissues, more particularly if the margin is involved, or else there will be a traumatic coloboma and an annoying epiphora. Black silk or harelip pin sutures are indicated, and the first suture should be at the lid margin. If not seen till twenty-four hours or more after the wound is produced, the edges must be pared to get healing by first intention.

Wounds of the conjunctiva alone are generally caused by some corroding agent, as acids or alkalies, and offer a favorable or unfavorable prognoisis depending directly on the extent of cornea involvement. If seen in time, wash out the offending agent, and, if that be an acid or alkaline substance, use at once an application that will neutralize or render it insoluble, after which it may be removed as a foreign body. Atropmize the iris, subdue the inflammation with cold applications and keep a soothing oleaginous substance applied until the resulting eschar is exfoliated, after which an antiseptic lotion with one of the following ointments will be indicated: Europhin grains two, aristol grains fifteen, or, iodoform grains ten, to petrolatum one drachm. If both the ocular and palpebral conjunctivæ are involved there is imminent danger of the two raw surfaces becoming united by the resulting cicatrix binding the lid to the eyeball, producing what we know as symblepharon. To obviate this condition of things the lids must be repeatedly separated from the ball and an antiseptic gauze kept interposed till healing is complete.

Superficial wounds of the cornea are among the most frequent of accidents, and are caused by the deposition in its structure of small pieces of steel, emery, grains of sand, cinders from a railroad locomotive, etc. The symptoms are, photophobia, lachrymation, blepharospasm and particularly severe pain, with more or less irritation of the iris. With a five per cent. solution of cocaine anæsthetize the parts, and, under focal illumination, lift out the offending body with a specially shaped needle or corneal spud. In mild cases little else will be needed save an irrigation of the conjunctival sac, with an antiseptic solution and a light pressure bandage during twelve to twenty-four hours, when the epithelium will have been reformed to protect the sensitive nerve endings. In more severe cases with ciliary or iritic irritation use atropia or scopolamine to paralyze the accomodation, and, as it were, put the iris in a splint.

The prognosis in this class of wounds is good. Incised, lacerated or punctured wounds of the cornea are more baneful in their results. There is usually an escape of aqueous followed by a prolapse of the iris, its fibres being entangled in the lips of the wound. If only the corneal tissue is impaired, with a prolapsed iris, we may preserve the integrity of the parts, provided we see it early and the wound does not become infected. First, we assure ourselves that no foreign body remains in the eye. Second, we endeavor to disengage the iris from its corneal incarceration by careful manipulation with the spatula, assisted by an aqueous solution of atropia, four grains to the ounce, if the rent be near the pupilary opening; and, with eserine, one grain to the ounce, if it be near its peripheral attachment. If we fail in this, as we most likely will do unless seen very soon after the accident, the portion prolapsed is to be grasped with a pair of delicate iris forceps, gently drawn out and as much of it as possible clipped off with scissors, after which the stump may be disengaged by the assistance of a mydriatic or myotic as the case demands. The conjunctival sac is then flushed with an antiseptic solution, the wound covered with finely pulverized iodoform, the lids gently closed and a light pressure bandage applied. If the lens or its capsule be punctured, and, although the iris remains intact and the cornea sustains only slight injury, there will be absorption of aqueous humor eventuating in traumatic cataract. If the agent inflicting the injury remain in the eye our first effort should be directed to its complete removal, for, without this the destruction of the eye by plastic iridocyclitis is almost certain to follow and a large per cent. are lost even with such removal. If the offending agent be located in the anterior chamber, the iris, or lens, we remove it through the original opening with forceps, or in the case of steel with the electro-magnet, if possible, or, failing in this, we may make a suitable opening for the purpose.

It must not be forgotten that wounds of the cornea involving its integrity beyond Bowman's membrane will be followed by a permanent opacity more or less dense and an impairment of the eye's function by a diffusion of rays of light directly in proportion to their density and situation in the pupilary area of the field of vision.

Wounds of the sclerotic, while less frequent than those of the cornea, are not rare. If there is a prolapse of vitreous it is to be clipped off with scissors, the opening in the sclerotic closed by fine silk sutures, the eye dressed antiseptically and favorable or unfavorable results expected according to the situation and extent of tissue involved. If the lesion be in the zone of danger and extend to, and include the ciliary body, the eye will almost certainly be lost by iridocyclitis and panophthalmitis. It is hardly necessary to say that in all manipulations of the wounded eye, either instrumental or otherwise, the most scrupulous antiseptic precautions should be rigorously observed.

As to those cases in which enucleation is indicated, no definite rule can be formulated, since the intelligence, occupation, and situation of the patient from the surgeon, must be taken into consideration. Of course, the object of excision is to remove the peril of sympathetic inflammation.

It is fortunate that sympathetic irritation, which is to be clearly differentiated from sympathetic inflammation, usually precedes the latter and serves as a signal for immediate enucleation. A wound involving the integrity of the zone of danger or one in which a foreign body remains in the eye, is the most prolific cause of sympathetic disturbances.

The concensus of opinion of the best authorities endorses the observations of Deutschmann that sympathetic ophthalmitis is of bacterial origin, the micro-organisms traveling by continuity of surface beneath the optic nerve sheath by way of the optic chiasm from the offending eye to the sympathizing member. The time at which sympathetic inflammation usually begins is from four to eight weeks after the traumatism, no case being on record as having occurred sooner than two weeks (Fuchs), while a case has been seen sixty years afterwards (Alt).

Sympathetic irritation is purely functional and has for its symptoms: lachrymation, blepharospasm, neuralgic pain in the supraorbital region, exquisite tenderness on pressure over the ciliary region, photophobia and impaired accomodation, the latter two being the more constant. When this condition of things obtains, immediate enucleation of the offending eye is the correct thing to do, and, the surgeon who fails to appreciate the situation and advise his patient accordingly falls far short of accomplishing his mission, inasmuch as a prompt removal of the source of irritation at this stage of the trouble will, in most cases, bring about a speedy subsidence of all symptoms and a pretention of the normal acuity of vision. If, however, from any cause enucleation has been deferred till sympathetic irritation has given place to well-marked symptoms of inflammation it should by no means be performed, provided any degree of vision remain in the offending eye, because in the end it will most likely prove to be the more useful of the two. Hence, prophylaxis, by enucleation of the offending eye before sympathetic ophthalmitis is precipitated in its fellow, is the only successful treatment. Therapeutically, we may use atropia, hot fomentations, local blood-letting by leeches to the temple, setons in chronic forms, scleral incisions if the intraocular tension be great and subconjunctival injections of hydrargarum bichloridum together with confinement in a dark room; but nothing heretofore practiced has been of much avail in the treatment of a well-developed case of sympathetic ophthalmia. A safe working rule with few exceptions demands enucleation: (1) When the wound is of such a nature as to destroy sight completely; (2) when the wound is in the ciliary region, especially if the iris be prolapsed, rendering inflammation reasonably certain; (3) when judicious efforts have failed to remove a foreign body and there is an iritis, even though vision be not entirely destroyed; and (4) when the offending eye is blind, to remove a source of irritation and thus render treatment of an existing sympathetic ophthalmitis more effectual.

The prognosis of sympathetic inflammation is hence very grave, and the surgeon should not fail to so inform the patient, or his friends, and if an attempt be made to save the offending eye, it should be done after a thorough understanding of the risk assumed as to sympathetic involvement. As a substitute for excision, evisceration and optico-ciliary neurotomy have been advocated, and practiced by some; but the consensus of professional opinion still adheres to enucleation as the safest line of procedure.

